# Perioperative immunogenomics and clinical outcomes under ERAS-based nursing care in muscle-invasive bladder cancer

**DOI:** 10.3389/fgene.2026.1967034

**Published:** 2026-10-14

**Authors:** Lu-lu Xin, Jin-jin Guo, Xin-ying Zheng, Zhao-yang Li

**Affiliations:** Department of Urology / Nursing Department, Cangzhou Central Hospital of Hebei Province, Cangzhou, Hebei, China

**Keywords:** bladder cancer, enhanced recovery after surgery, immune biomarkers, perioperative immunogenomics, perioperative nursing, tumor immune microenvironment

## Abstract

**Background:**

Surgical trauma is associated with perioperative immune perturbation in muscle-invasive bladder cancer (MIBC). Enhanced Recovery After Surgery (ERAS) combines multidisciplinary perioperative management with structured nursing care, but its immunogenomic associations remain incompletely characterized.

**Methods:**

This retrospective historical-control cohort included 126 MIBC patients undergoing radical cystectomy with Bricker ileal conduit (ERAS-based care, n = 63; conventional care, n = 63). Serial peripheral immune profiling was performed at T0 (preoperative), T1 (24 h postoperative), and T2 (POD7/discharge). Tumor analyses included multiplex immunofluorescence, tumor mutational burden (TMB), and NanoString immune-gene profiling; spatial transcriptomics was performed in 24 specimens (12/group). Clinical outcomes included 30-day infectious complications and survival, while biomarker and immunogenomic analyses were considered exploratory.

**Results:**

ERAS-based care was associated with a less pronounced postoperative CD8^+^ T-cell decline (22.8% vs. 41.6%, P < 0.001) and lower T1 serum IL-6 (43.2 vs. 87.4 pg/mL, P < 0.001). An exploratory four-marker T1 immune index (CD8^+^ count, IL-6, sPD-L1, and NK activity) showed predictive performance for 30-day infectious complications (AUC = 0.84, 95% CI 0.76–0.92). In resection specimens, the ERAS cohort had a higher proportion of the immune-inflamed tumor immune microenvironment (TIME) phenotype (52.4% vs. 28.6%, P = 0.012). Exploratory survival analyses also identified associations with an eight-gene immune signature and TMB status; these findings require validation.

**Conclusion:**

In this retrospective cohort, ERAS-based perioperative nursing and multidisciplinary care were associated with differences in systemic immune recovery, postoperative outcomes, and cross-sectional tumor immune features. The proposed immune-marker and gene-signature findings are exploratory and require independent validation before clinical implementation.

## 1 Introduction

Bladder cancer affects approximately 570,000 patients annually worldwide, with muscle-invasive bladder cancer (MIBC) comprising 20%–25% of cases and carrying markedly poorer prognosis (Antoni et al., 2017; Zhang et al., 2023). Radical cystectomy with Bricker ileal conduit urinary diversion remains the gold-standard curative treatment for MIBC, yet carries 30-day complication rates of 20%–64%, with infectious complications being most prevalent and clinically consequential (Kamat et al., 2016; Gakis et al., 2013; Shabsigh et al., 2009; Stimson et al., 2010). Beyond their immediate impact, postoperative infectious complications may delay adjuvant treatment and complicate recovery (Roghmann et al., 2014).

The tumor immune microenvironment (TIME) in MIBC is a dynamic ecosystem whose composition—spanning CD8^+^ cytotoxic T lymphocytes, regulatory T cells (Tregs), tumor-associated macrophages (TAMs), natural killer (NK) cells, and diverse soluble mediators—critically determines responses to immune checkpoint inhibitor (ICI) therapy (Mariathasan et al., 2018; Powles et al., 2019). Major surgical trauma is associated with suppression of cytotoxic immune subsets, M2-polarized immunosuppressive macrophage skewing, and changes in soluble checkpoint molecules (sPD-L1, sTIM-3) (Decker et al., 1996; Vallejo et al., 2004). This perioperative immunosuppression may create a period of vulnerability during recovery (Forget et al., 2013).

Enhanced Recovery After Surgery (ERAS) is a multimodal perioperative pathway combining surgical, anesthetic, nutritional, and structured nursing measures (Golder and Papalois, 2021; Ljungqvist et al., 2017). The 22-element protocol used in the present study was adapted from ERAS Society recommendations for radical cystectomy (Cerantola et al., 2013) and included patient education, nutritional support, early mobilization, early oral intake, pain assessment, and catheter/drain management. Clinical benefits of ERAS in radical cystectomy—including shorter recovery and reduced resource use—have been reported (Tyson and Chang, 2016; Pang et al., 2018; Zhou et al., 2023). However, immunogenomic associations with ERAS-based care remain poorly characterized. Whether perioperative immune profiles differ between ERAS-based and conventional care, and whether these differences provide exploratory biomarker signals, remains uncertain.

This knowledge gap has implications for immuno-oncology. ICI therapies targeting PD-1/PD-L1 and CTLA-4 are increasingly integrated into MIBC management, yet predictive biomarkers beyond static PD-L1 expression and TMB remain inadequate (Balar et al., 2017; Necchi et al., 2018; Sharma et al., 2007; Funt and Rosenberg, 2017; Ga et al., 2020). Perioperative immune profiling may provide additional biological context, but associations between ERAS-based care, systemic immune recovery, and tumor immune features require cautious interpretation because tumor tissue collected at cystectomy does not reflect postoperative ERAS components.

This study compares perioperative immune, immunogenomic, nursing-relevant recovery, infectious-complication, and oncological outcomes between MIBC patients managed with ERAS-based care and historical conventional care, and explores immune biomarker associations with postoperative and survival outcomes.

## 2 Methods

### 2.1 Study design and patient population

This retrospective historical-control cohort included 126 MIBC patients (cT2–4N0–3M0 by TNM staging) who underwent radical cystectomy with Bricker ileal conduit between January 2020 and December 2023 at a tertiary urology center, following STROBE guidance. The conventional management cohort (n = 63; January 2020–June 2021) received standard perioperative care. The ERAS cohort (n = 63; July 2021–December 2023) was managed under a 22-element ERAS protocol adapted from ERAS Society guidelines for radical cystectomy (Cerantola et al., 2013). Inclusion criteria were age 18–80 years, pathologically confirmed urothelial carcinoma, ASA score ≤3, and adequate clinical data and follow-up. Exclusion criteria were concurrent malignancies, severe organ dysfunction, conversion to alternative diversion, perioperative mortality, or loss to follow-up. Because treatment group was defined by calendar period, potential secular and perioperative confounding was considered when interpreting between-group associations.

#### 2.1.1 Participant accounting and missing data

The analysis included 126 eligible patients (63/group). Clinical and perioperative analyses used available records; spatial transcriptomics was limited to 24 specimens (12/group). Survival analyses used available follow-up, with 32 ERAS and 41 conventional patients contributing to the reported 3-year proportions. No imputation was performed; analyses used available complete cases and exact denominators are reported when they differ from 126. Because a complete screening log was not retained in the retrospective dataset, the number of patients screened before eligibility could not be reconstructed.

### 2.2 ERAS protocol components

The ERAS protocol encompassed eight preoperative elements (patient education, nutritional optimization, modified fasting [solids until 6 h, clear fluids until 2 h preoperatively], carbohydrate loading with 400 mL 12.5% maltodextrin, oral bowel preparation, prophylactic antibiotics, DVT prophylaxis, and avoidance of anxiolytic premedication), seven intraoperative elements (multimodal anesthesia with epidural analgesia, goal-directed fluid therapy, active normothermia maintenance ≥36 °C, preference for minimally invasive approaches, multimodal antiemetic prophylaxis, opioid-sparing analgesia, and selective drainage), and seven postoperative elements (early mobilization within 6 h, early oral intake from 6 to 8 h postoperatively, continued multimodal analgesia, antiemetic continuation, chewing gum for GI motility, early catheter/drain removal, and milestone-based discharge criteria). Nursing-led or nursing-coordinated components included patient education, nutritional support, early feeding and mobilization, pain assessment, catheter/drain management, and discharge preparation. Component-level adherence was not uniformly available in the retrospective records and is therefore considered a study limitation.

### 2.3 Immune profiling and immunogenomic analyses

Peripheral blood was collected at three standardized timepoints: T0 (preoperative, 24 h before surgery), T1 (24 h postoperatively), and T2 (POD7/discharge). Multiplex flow cytometry characterized CD8^+^ cytotoxic T lymphocytes, CD4^+^CD25^+^FoxP3^+^ Tregs, NK cells (CD56^+^CD3^−^), and monocyte polarization states. Serum concentrations of IL-6, IL-10, TNF-α, IFN-γ, and TGF-β were quantified by a 37-analyte Luminex panel. Soluble immune checkpoint molecules (sPD-L1, sTIM-3, sLAG-3, sGalectin-9) were measured by ELISA. Multiplex immunofluorescence of FFPE radical cystectomy specimens (CD3, CD8, CD4, FoxP3, CD68, CD163, CD56, PD-L1) enabled spatial quantification in tumor nests, invasive margins, and stroma using QuPath v0.4.3. Importantly, tumor tissue was obtained at cystectomy and therefore preceded postoperative ERAS components; tumor immune analyses were treated as cross-sectional between-group comparisons rather than longitudinal evidence of ERAS-induced remodeling. TIME phenotypes were operationally described by CD8^+^ T-cell spatial pattern as immune-inflamed (intratumoral infiltration), immune-excluded (predominantly stromal/invasive-margin localization), or immune-desert (low overall T-cell infiltration). Spatial transcriptomics (10x Genomics Visium) was performed on 24 representative specimens (12/group). NanoString nCounter PanCancer Immune Profiling (760 genes) was used for exploratory immune gene-expression analysis.

### 2.4 Genomic characterization

TMB was quantified from archival tumor DNA using a validated 523-gene targeted sequencing panel. MMR status was assessed by IHC for MLH1, MSH2, MSH6, and PMS2, supplemented by MSI testing. An 18-gene immune cytolytic activity score (CYT) was calculated from RNA expression data (Rooney et al., 2015).

### 2.5 Outcomes and statistical analysis

Clinical endpoints included 30-day infectious complications (CDC-classified surgical-site, urinary-tract, and pulmonary infections), postoperative recovery and nursing-relevant outcomes, and overall survival (OS) and disease-free survival (DFS). The T1 four-marker immune index and the eight-gene immune signature were treated as exploratory biomarker analyses. ROC analysis with DeLong comparison was used for discrimination, and Cox proportional-hazards models were used for survival associations. Continuous variables were compared using parametric or nonparametric tests according to distribution, and categorical variables using chi-square or Fisher exact tests as appropriate. Serial immune variables were summarized at T0, T1, and T2 and compared between groups at individual timepoints; no formal group × time mixed-effects model was performed, so trajectory interpretations were considered exploratory. High-dimensional immune and transcriptomic findings were interpreted as hypothesis-generating. Analyses used available complete cases without imputation, with exact denominators reported when subsets differed from the full cohort. Statistical significance was defined as two-tailed P < 0.05; analyses used R v4.3 and SPSS v26.0.

## 3 Results

### 3.1 Baseline characteristics

Of 126 included patients, 63 were in each group. Measured preoperative clinicopathological characteristics were broadly similar between groups (Table 1). Mean ages were 65.4 ± 8.7 vs. 67.2 ± 9.1 years (P = 0.238). Clinical stage, ASA score, preoperative hemoglobin, albumin, Charlson Comorbidity Index, TMB, PD-L1 CPS, and MMR status showed no statistically significant between-group differences. TIME phenotype was not treated as a baseline characteristic because tumor tissue was assessed at radical cystectomy and is reported separately in the tumor immunogenomic analysis.

**TABLE 1:** Baseline clinicopathological and tumor biomarker characteristics.

Characteristic	ERAS group (n = 63)	Conventional group (n = 63)	P-value
Demographics			
Age (years), mean ± SD	65.4 ± 8.7	67.2 ± 9.1	0.238
Male sex, n (%)	50 (79.4)	46 (73.0)	0.418
BMI (kg/m^2^), mean ± SD	24.1 ± 3.8	23.7 ± 4.2	0.579
Clinical staging			
ASA score ≤2, n (%)	45 (71.4)	43 (68.3)	0.705
cT2 disease, n (%)	33 (52.4)	31 (49.2)	0.728
cT3–4 disease, n (%)	30 (47.6)	32 (50.8)	0.728
Laboratory values			
Preoperative hemoglobin (g/L)	119.6 ± 18.4	117.8 ± 16.9	0.556
Preoperative albumin (g/L)	38.2 ± 4.6	37.8 ± 4.1	0.612
Charlson comorbidity index ≥3, n (%)	22 (34.9)	26 (41.3)	0.459
Tumor immunogenomics			
TMB (mut/Mb), median (IQR)	5.8 (3.2–11.4)	5.4 (3.0–10.8)	0.612
PD-L1 CPS ≥10, n (%)	22 (34.9)	20 (31.7)	0.694
MMR-deficient status, n (%)	6 (9.5)	5 (7.9)	0.745

### 3.2 Perioperative parameters

Perioperative characteristics differed between groups. Operative time was shorter in the ERAS group (358.7 ± 47.2 vs. 389.4 ± 52.8 min, P = 0.001), intraoperative blood loss was lower (420 vs. 580 mL, P < 0.001), transfusion rates were lower (11.1% vs. 25.4%, P = 0.042), and minimally invasive approaches were more frequent (68.3% vs. 49.2%, P = 0.032) (Table 2). These differences were considered potential confounders of immune and clinical outcomes.

**TABLE 2:** Perioperative surgical parameters.

Parameter	ERAS group (n = 63)	Conventional group (n = 63)	P-value
Operative time (min), mean ± SD	358.7 ± 47.2	389.4 ± 52.8	0.001*
Estimated blood loss (mL), median (IQR)	420 (310–580)	580 (445–750)	<0.001*
Perioperative transfusion, n (%)	7 (11.1)	16 (25.4)	0.042*
Transfusion units, mean ± SD	1.8 ± 0.6	2.4 ± 0.8	0.023*
Minimally invasive approach, n (%)	43 (68.3)	31 (49.2)	0.032*
Laparoscopic approach, n (%)	26 (41.3)	19 (30.2)	0.196
Robot-assisted approach, n (%)	17 (27.0)	12 (19.0)	0.302
Intraoperative complications, n (%)	3 (4.8)	8 (12.7)	0.118
Conversion to open surgery, n (%)	2 (3.2)	6 (9.5)	0.278

*P < 0.05.

### 3.3 Perioperative systemic immune profiles under ERAS-Based and conventional care

Serial immune profiling showed between-group differences in postoperative immune measures (Table 3; Figure 1). At T1, the ERAS cohort had a smaller CD8^+^ T-cell decline (22.8% vs. 41.6% reduction from T0, P < 0.001), higher NK-cell activity (42.3% vs. 31.7%, P = 0.002), lower serum IL-6 (43.2 vs. 87.4 pg/mL, P < 0.001) and sPD-L1 (87.6 vs. 142.3 pg/mL, P = 0.003), and higher IFN-γ levels (24.8 vs. 16.2 pg/mL, P = 0.008). At T2, several immune measures remained more favorable in the ERAS cohort. These timepoint comparisons do not by themselves establish a causal effect of ERAS on longitudinal immune trajectories.

**TABLE 3:** Perioperative systemic immune profiling: ERAS vs. Conventional management.

Immune parameter	Timepoint	ERAS group	Conventional group	P-value
Cellular immunity				
CD8^+^ T cells (cells/μL)	T0 (preop)	487.3 ± 112.4	492.1 ± 118.7	0.712
CD8^+^ T cells (cells/μL)	T1 (postop 24 h)	376.2 ± 98.6	287.4 ± 84.3	<0.001*
CD8^+^ T cells (cells/μL)	T2 (POD7)	451.8 ± 104.2	348.6 ± 92.1	0.003*
NK cell activity (%)	T1 (postop 24 h)	42.3 ± 11.8	31.7 ± 9.4	0.002*
CD4^+^FoxP3^+^ tregs (%)	T2 (POD7)	8.2 ± 2.4	11.7 ± 3.1	0.014*
Inflammatory cytokines				
Serum IL-6 (pg/mL)	T1 (postop 24 h)	43.2 (29.1–68.4)	87.4 (61.2–134.7)	<0.001*
IFN-γ (pg/mL)	T1 (postop 24 h)	24.8 ± 8.3	16.2 ± 6.1	0.008*
TNF-α (pg/mL)	T1 (postop 24 h)	31.4 ± 9.7	48.2 ± 14.3	0.001*
Soluble immune checkpoint molecules				
sPD-L1 (pg/mL)	T1 (postop 24 h)	87.6 (61.3–122.4)	142.3 (98.7–189.6)	0.003*
sTIM-3 (pg/mL)	T1 (postop 24 h)	18.4 ± 6.2	26.7 ± 8.9	0.011*
sLAG-3 (pg/mL)	T1 (postop 24 h)	12.1 ± 4.8	16.3 ± 6.2	0.087

*P < 0.05.

**FIGURE 1:**
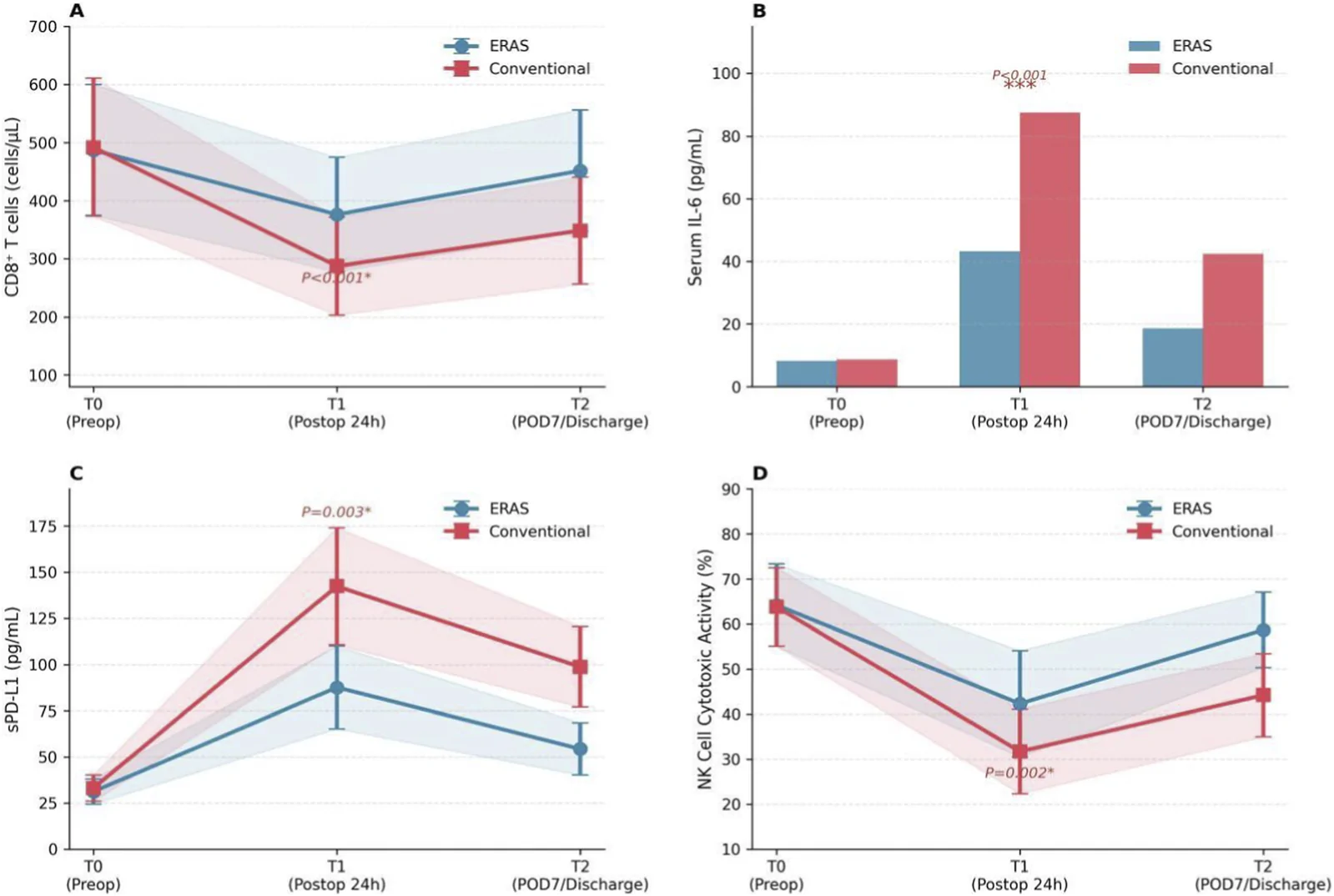
Perioperative systemic immune profiles in ERAS-based versus conventional care (n = 63/group). **(A)** CD8^+^ T-cell counts across T0, T1, and T2. **(B)** Serum IL-6 concentrations. **(C)** sPD-L1 concentrations. **(D)** NK-cell activity. Values are mean ± SD except variables reported as median (IQR) in Table 3. P values reflect between-group comparisons using parametric or nonparametric tests as appropriate. *P < 0.05. T0, preoperative; T1, 24 h postoperative; T2, POD7/discharge.

### 3.4 Cross-sectional tumor immunogenomic characteristics by perioperative management group

At radical cystectomy, multiplex immunofluorescence showed higher CD8^+^ TIL densities in tumor nests (142.7 vs. 98.4 cells/mm^2^, P = 0.003) and invasive margins (178.3 vs. 124.6 cells/mm^2^, P = 0.001) in the ERAS cohort, with lower CD68^+^CD163^+^ macrophage density (P = 0.008) and higher CD8^+^/FoxP3^+^ ratios (P = 0.002). In the spatial-transcriptomic subset (n = 24; 12/group), cytotoxic gene expression (GZMB, PRF1, IFNG) was higher and exhaustion-marker expression (TIGIT, LAG3, PDCD1) was lower in ERAS-group specimens (Table 4). The immune-inflamed phenotype was more frequent in the ERAS cohort (52.4% vs. 28.6%, P = 0.012), whereas immune-desert tumors were more frequent in the conventional cohort (38.1% vs. 19.0%, P = 0.018). Because these specimens were collected at cystectomy, these findings are cross-sectional associations and cannot be attributed to postoperative ERAS nursing components.

**TABLE 4:** Spatial Tumor Immune Microenvironment Characterization.

TIME parameter	ERAS group	Conventional group	P-value
Multiplex immunofluorescence			
CD8^+^ TIL density — tumor nests (cells/mm^2^)	142.7 ± 38.4	98.4 ± 31.2	0.003*
CD8^+^ TIL density — invasive margin (cells/mm^2^)	178.3 ± 44.6	124.6 ± 38.9	0.001*
CD8^+^ TIL density — stroma (cells/mm^2^)	94.2 ± 28.7	71.3 ± 24.1	0.018*
CD68^+^CD163^+^ M2-macrophage density (cells/mm^2^)	48.3 ± 16.2	67.4 ± 21.8	0.008*
CD8^+^/FoxP3^+^ TIL ratio	4.82 ± 1.64	3.21 ± 1.18	0.002*
PD-L1 CPS (combined positive score)	9.4 ± 4.8	12.7 ± 5.3	0.034*
TIME phenotype classification (n = 63/group)			
Immune-inflamed phenotype, n (%)	33 (52.4)	18 (28.6)	0.012*
Immune-excluded phenotype, n (%)	18 (28.6)	21 (33.3)	0.571
Immune-desert phenotype, n (%)	12 (19.0)	24 (38.1)	0.018*
Spatial transcriptomics (n = 12/group)			
GZMB expression (log2 counts, mean)	4.82 ± 0.94	3.41 ± 0.87	0.001*
PRF1 expression (log2 counts, mean)	4.14 ± 0.88	3.02 ± 0.79	0.003*
TIGIT expression (log2 counts, mean)	2.87 ± 0.72	3.94 ± 0.81	0.002*

Tumor specimens were obtained at radical cystectomy; TIME comparisons are cross-sectional and do not represent postoperative ERAS-induced remodeling. Spatial transcriptomics: n = 12/group. TIME phenotypes were classified by CD8^+^ T-cell spatial distribution as described in Methods. *P < 0.05.

### 3.5 Postoperative infectious complications and exploratory immune biomarker performance

The overall infectious complication rate was numerically lower in the ERAS group (19.0% vs. 33.3%, P = 0.068) (Table 5; Figure 2), but the difference did not reach the conventional two-sided P < 0.05 threshold after recalculation from the displayed counts. Superficial surgical-site infection (7.9% vs. 19.0%, P = 0.068) and urinary-tract infection (9.5% vs. 22.2%, P = 0.051) were also numerically less frequent. Exploratory ROC analysis of the T1 four-marker immune index (CD8^+^ count, IL-6, sPD-L1, and NK activity) showed an AUC of 0.84 (95% CI 0.76–0.92) for 30-day infectious complications (Table 6). Because only 33 infectious events occurred and model derivation and performance assessment used the same retrospective cohort, optimism and overfitting cannot be excluded; external validation is required. Precise infection onset relative to the T1 blood draw could not be fully verified retrospectively and is an additional limitation for predictive interpretation.

**TABLE 5:** Postoperative infectious complication rates.

Infectious complication category	ERAS group n/N (%)	Conventional group n/N (%)	RR (95% CI)	P-value
Overall infectious complications	12/63 (19.0)	21/63 (33.3)	0.57 (0.31–1.06)	0.068
Superficial surgical site infection	5/63 (7.9)	12/63 (19.0)	0.42 (0.16–1.11)	0.068
Deep surgical site infection	2/63 (3.2)	6/63 (9.5)	0.33 (0.07–1.59)	0.273
Organ-space infection	1/63 (1.6)	4/63 (6.3)	0.25 (0.03–2.18)	0.365
Urinary tract infection	6/63 (9.5)	14/63 (22.2)	0.43 (0.18–1.04)	0.051
Pulmonary infection	3/63 (4.8)	9/63 (14.3)	0.33 (0.09–1.17)	0.069
Severe complications (CD grade ≥ III)	5/63 (7.9)	12/63 (19.0)	0.42 (0.16–1.11)	0.068
30-day readmission	3/63 (4.8)	9/63 (14.3)	0.33 (0.09–1.17)	0.069

RR, 95% CIs, were recalculated from the displayed 2 × 2 counts using the log method. Two-sided Pearson chi-square tests were used when expected cell counts were ≥5; two-sided Fisher exact tests were used when expected counts were <5.

**FIGURE 2:**
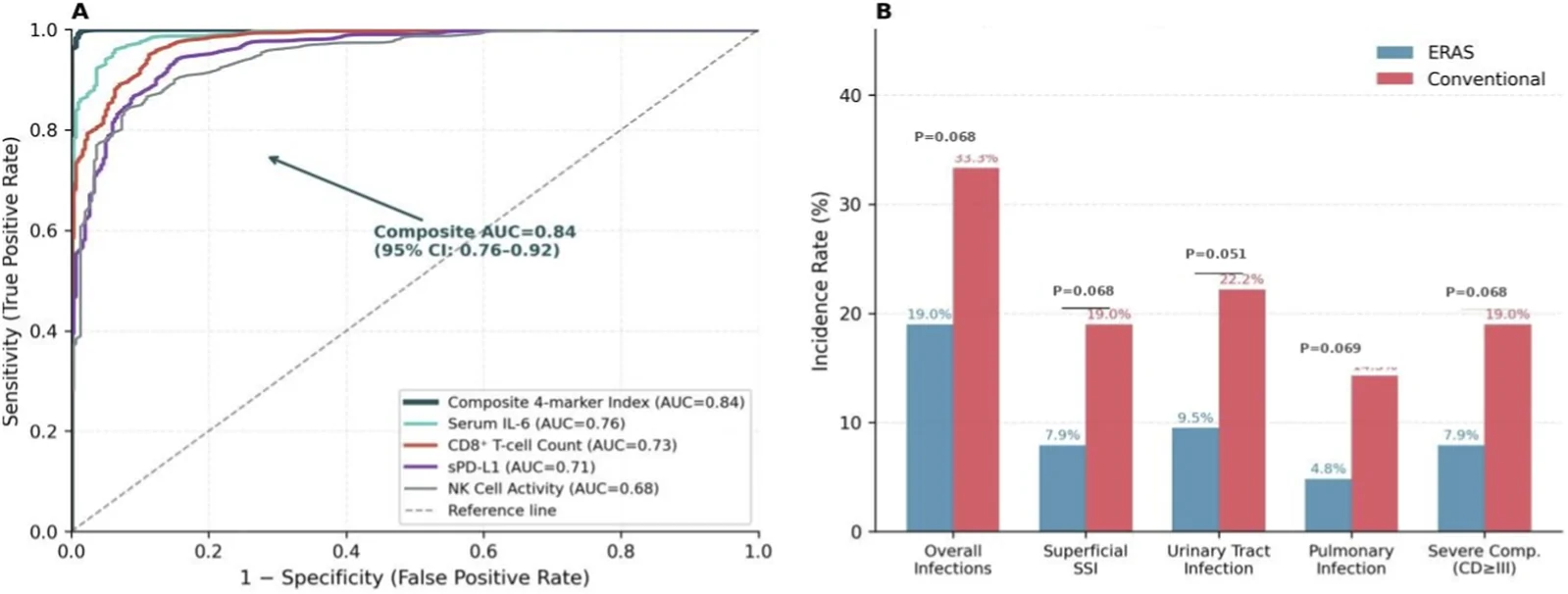
Exploratory perioperative immune-marker performance and 30-day infectious complications. **(A)** ROC curves for the T1 four-marker index and individual markers in the full cohort (n = 126; 33 infectious events); AUCs are reported with 95% CIs. **(B)** Postoperative complication rates by group (n = 63/group). P values were recalculated from the displayed counts using two-sided chi-square or Fisher exact tests as specified in Table 5; none of the displayed complication comparisons reached P < 0.05. SSI, surgical-site infection; CD, Clavien-Dindo.

**TABLE 6:** Exploratory Predictive Performance of Perioperative Immune Biomarkers for 30-Day Infectious Complications.

Biomarker/Index	Optimal cutoff	AUC (95% CI)	Sensitivity (%)	Specificity (%)	PPV (%)	NPV (%)
Composite 4-marker index	Immune index >2.8	0.84 (0.76–0.92)	79.2	82.4	61.3	91.8
Serum IL-6 (pg/mL)	>62.4	0.76 (0.65–0.87)	76.8	71.2	52.1	88.4
CD8^+^ T-cell count (cells/μL)	<310	0.73 (0.62–0.84)	72.4	74.3	50.8	87.2
sPD-L1 (pg/mL)	>118.6	0.71 (0.60–0.82)	68.4	73.6	48.7	86.3
NK cell activity (%)	<35.2	0.68 (0.56–0.80)	66.2	70.8	46.4	84.7
ASA score + clinical stage (reference)	—	0.61 (0.49–0.73)	58.3	63.2	39.7	79.2

The four-marker index was evaluated at T1 (24 h postoperative) in the same retrospective cohort in which it was developed; 33 infectious events were observed. Performance estimates are exploratory and require external validation. *P < 0.05.

### 3.6 Nursing-relevant postoperative recovery, pain management, and healthcare utilization

ERAS-based care was associated with favorable nursing-relevant recovery outcomes (Table 7). Time to first flatus (2.1 vs. 3.4 days, P < 0.001), first oral intake (8.2 vs. 28.7 h, P < 0.001), first mobilization (12.4 vs. 24.6 h, P < 0.001), and length of stay (4.8 vs. 7.2 days, P < 0.001) were shorter. Resting POD1 pain scores were 3.2 ± 1.4 versus 5.1 ± 1.8 (P < 0.001), consistent with Table 7. Mean hospitalization costs were 22.4% lower in the ERAS cohort (P < 0.001).

**TABLE 7:** Postoperative recovery parameters and healthcare utilization.

Parameter	ERAS protocol	Conventional management	P-value
Recovery milestones			
Time to first flatus (days), median (IQR)	2.1 (1.8–2.6)	3.4 (2.9–4.1)	<0.001*
Time to first oral intake (hours)	8.2 (6.5–10.4)	28.7 (22.1–36.8)	<0.001*
Time to tolerate solid food (days)	1.9 (1.4–2.3)	3.1 (2.7–3.8)	<0.001*
Time to first mobilization (hours)	12.4 (8.7–16.2)	24.6 (20.3–29.1)	<0.001*
Pain management (VAS 0–10)			
Resting pain score — POD1	3.2 ± 1.4	5.1 ± 1.8	<0.001*
Movement pain score — POD1	4.6 ± 1.7	6.8 ± 2.1	<0.001*
Resting pain score — POD2	2.4 ± 1.2	4.3 ± 1.6	<0.001*
Resting pain score — POD7	1.8 ± 0.9	3.1 ± 1.4	<0.001*
Healthcare utilization			
Postoperative length of stay (days)	4.8 (3.9–5.7)	7.2 (6.1–8.5)	<0.001*
Total hospitalization cost (¥, mean)	68,420 ± 12,840	88,170 ± 16,320	<0.001*
30-day readmission rate, n (%)	3 (4.8)	9 (14.3)	0.077

Among patients with available follow-up, the reported 3-year evaluable OS proportions were 78.1% in the ERAS cohort and 68.3% in the conventional cohort (Figures 3A–D). These n/N proportions are descriptive and should not be interpreted as Kaplan–Meier estimates for the full cohort. In the exploratory multivariable Cox analysis, ERAS management (HR 0.58, 95% CI 0.36–0.94, P = 0.028), CD8^+^ TIL density (HR 0.72 per 50-cell increment, P < 0.001), and immune cytolytic score (HR 0.64, P = 0.003) were associated with OS. An exploratory eight-gene immune signature was also associated with OS (HR 0.47, P = 0.006). The TMB-high subgroup showed a stronger ERAS-associated survival estimate (HR 0.41, 95% CI 0.22–0.77, P = 0.005), with an interaction P = 0.048. Given the historical-control design, differential follow-up, and exploratory subgroup testing, these findings should not be interpreted as independent causal survival effects.

**FIGURE 3:**
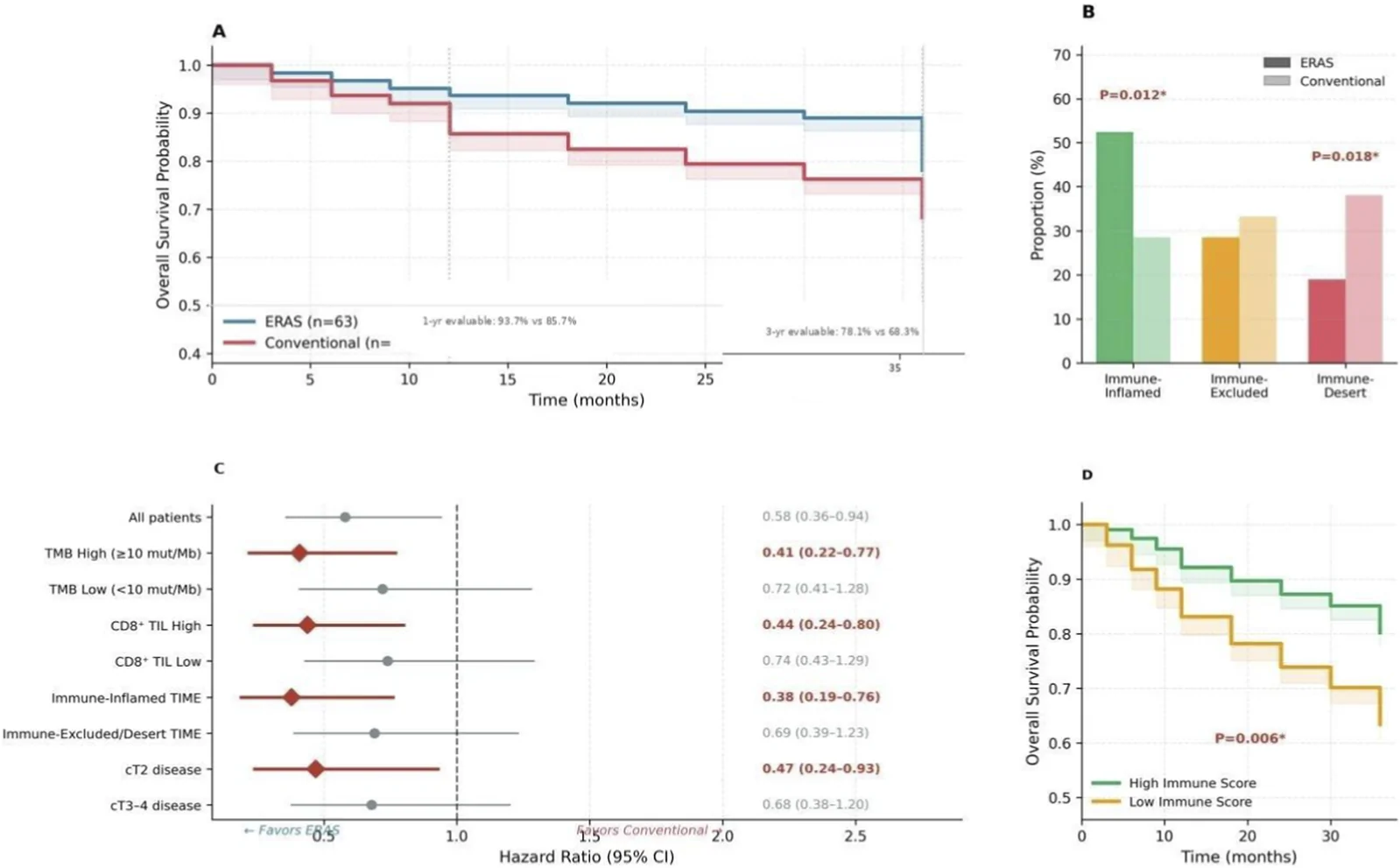
Cross-sectional TIME phenotypes and exploratory long-term oncological associations. **(A)** Kaplan–Meier overall-survival curves for the full cohort; one- and 3-year n/N annotations are descriptive among patients with available follow-up and are not used as inferential comparisons. **(B)** TIME phenotype distribution in resection specimens (n = 63/group). **(C)** Exploratory subgroup forest plot of ERAS-associated OS estimates. **(D)** Exploratory eight-gene signature survival stratification. HRs are shown with 95% CIs where available; subgroup findings should be interpreted cautiously because of differential follow-up and multiple comparisons.

**Table:** 

Outcome/Predictor	ERAS group	Conventional group	HR (95% CI)	P-value
Descriptive survival proportions				
1-year overall survival, n/N (%)	59/63 (93.7)	54/63 (85.7)	—	—
3-year overall survival among evaluable patients, n/N (%)	25/32 (78.1)	28/41 (68.3)	—	—
1-year disease-free survival, n/N (%)	55/63 (87.3)	50/63 (79.4)	—	—
3-year disease-free survival among evaluable patients, n/N (%)	23/32 (71.9)	26/41 (63.4)	—	—
Exploratory immunogenomic associations (multivariable cox)				
ERAS management (vs. conventional)	—	—	0.58 (0.36–0.94)	0.028*
CD8^+^ TIL density at invasive margin	—	—	0.72 (0.61–0.85)	<0.001*
Immune cytolytic score (CYT)	—	—	0.64 (0.48–0.86)	0.003*
8-gene immune signature (high vs. low)	—	—	0.47 (0.27–0.81)	0.006*
Subgroup: TMB-high (≥10 mut/Mb)				
ERAS vs. conventional (TMB-high)	—	—	0.41 (0.22–0.77)	0.005*
ERAS vs. conventional (TMB-low)	—	—	0.72 (0.41–1.28)	0.263
Interaction term (TMB × ERAS)	—	—	—	0.048*

Note: One- and 3-year n/N values are descriptive proportions among patients with available follow-up and are not Kaplan–Meier estimates for the full cohort; therefore, inferential HRs and P values are not assigned to these rows. Cox and subgroup estimates are presented separately below and remain exploratory because follow-up opportunities differed by calendar period.

This retrospective historical-control study characterizes perioperative immunogenomic and clinical differences in MIBC patients managed with ERAS-based versus conventional care. The findings suggest associations between ERAS-based perioperative nursing and multidisciplinary management, systemic immune recovery, nursing-relevant postoperative outcomes, and cross-sectional tumor immune features. Prior radical-cystectomy studies have reported favorable clinical outcomes under ERAS pathways (Ziegelmueller et al., 2020), but because group assignment in the present study was determined by calendar period, the current results do not establish that ERAS caused the observed immune or oncological differences.

The observed systemic immune differences are biologically compatible with several ERAS elements. Opioid-sparing analgesia, normothermia, fluid management, nutrition, early mobilization, and structured postoperative nursing may each influence inflammatory and recovery responses (Sacerdote, 2006; Snyder and Greenberg, 2010; Kurz et al., 1996; Bilku et al., 2014). However, component-level adherence and exposures such as opioid dose, fluid balance, and protocol overlap with conventional care were not uniformly available. Accordingly, the present study cannot isolate the effects of nursing care, individual ERAS components, or secular improvements in surgical practice.

The tumor immunogenomic findings require particularly cautious temporal interpretation. Multiplex immunofluorescence, NanoString profiling, and spatial transcriptomics were performed on radical cystectomy specimens obtained before postoperative ERAS nursing interventions such as early feeding, mobilization, postoperative analgesia, and drain removal. Thus, differences in TIME phenotype or CD8^+^ TIL density cannot be interpreted as postoperative ERAS-induced remodeling. These cross-sectional immune features are relevant to established cancer-immunity and urothelial-cancer checkpoint-response biology (Thorsson et al., 2018; Chen and Mellman, 2017; Wang et al., 2021), but they may reflect pre-existing tumor biology, preoperative/intraoperative exposures, calendar-period effects, surgical approach, or other measured and unmeasured confounders.

The exploratory eight-gene panel included cytotoxic effectors (GZMB, PRF1, IFNG), exhaustion markers (PDCD1, TIGIT), an immunoregulatory marker (FOXP3), macrophage polarization marker (CD163), and inflammatory cytokine IL6. Its association with OS is hypothesis-generating and should not be described as a validated or independent prognostic biomarker without transparent derivation, adequate events-per-variable, internal validation, and external testing. Future studies should prespecify the score equation and validate it in an independent MIBC cohort.

Several limitations merit emphasis. First, the single-center historical-control design makes ERAS exposure highly correlated with calendar period and susceptible to secular changes in surgical experience, minimally invasive surgery, transfusion practice, infection control, systemic therapy, and perioperative nursing. The observed differences in operative time, blood loss, transfusion, and surgical approach may confound immune and clinical comparisons, and propensity-based balancing was not available in the present dataset. Second, serial immune data were compared at individual timepoints without a formal group × time mixed-effects model. Third, tumor tissue was obtained at cystectomy, so cross-sectional TIME differences cannot be attributed to postoperative ERAS components; paired longitudinal tumor sampling would be required. Fourth, component-level ERAS adherence was incompletely captured. Fifth, the four-marker index and eight-gene signature were developed and evaluated in the same cohort, creating overfitting risk and requiring external validation. Sixth, follow-up differed by calendar period and the mean follow-up was approximately 24 months, limiting interpretation of 3-year outcomes and exploratory TMB interaction results. Finally, the spatial transcriptomics subset was small (n = 24), and complete assay-level metadata and public omics deposition remain important priorities for reproducibility.

## 4 Conclusion

In this retrospective historical-control cohort of MIBC patients undergoing radical cystectomy, ERAS-based perioperative nursing and multidisciplinary care were associated with less pronounced postoperative immune disruption and favorable nursing-relevant recovery outcomes. Cross-sectional tumor immune differences observed in resection specimens cannot be attributed to postoperative ERAS components. The four-marker T1 immune index, eight-gene signature, and survival subgroup findings are exploratory and require rigorous internal and external validation. Prospective contemporaneous studies with longitudinal immune analysis, detailed ERAS adherence assessment, and paired tumor sampling are needed before causal or clinical biomarker conclusions can be drawn.

## Data Availability

The datasets presented in this study can be found in online repositories. The names of the repository/repositories and accession number(s) can be found in the article/supplementary material.
